# Assessing the Accuracy of Large Language Models on European Guidelines for Cervical Cancer: An In Silico Benchmarking Study

**DOI:** 10.1111/1471-0528.70095

**Published:** 2025-11-24

**Authors:** Matteo Pavone, Chiara Innocenzi, Nicola Macellari, Chiara Cantarini, Massimo Criscione, Andrea Rosati, Lise Lecointre, Antonella Carcagnì, Barbara Costantini, Jacques Marescaux, Anna Fagotti, Francesco Fanfani, David Cibula, Denis Querleu, Nicolò Bizzarri

**Affiliations:** ^1^ UOC Ginecologia Oncologica, Dipartimento di Scienze per la Salute Della Donna e del Bambino e di Sanità Pubblica, Fondazione Policlinico Universitario A. Gemelli, IRCCS Rome Italy; ^2^ Research Institute Against Digestive Cancer, IRCAD Strasbourg Strasbourg France; ^3^ Institute of Image‐Guided Surgery, IHU Strasbourg Strasbourg France; ^4^ ICube, Laboratory of Engineering, Computer Science and Imaging, Department of Robotics, Imaging, Teledetection and Healthcare Technologies, University of Strasbourg, CNRS, UMR 7357 Strasbourg France; ^5^ Department of Gynecologic Surgery University Hospitals of Strasbourg Strasbourg France; ^6^ Facility of Epidemiology and Biostatistics ‐ Gemelli Generator, Fondazione Policlinico Universitario A. Gemelli IRCCS Rome Italy; ^7^ UniCamillus, International Medical University Rome Italy; ^8^ Università Cattolica del Sacro Cuore Rome Italy; ^9^ Department of Obstetrics and Gynecology First Faculty of Medicine, Charles University, General University Hospital in Prague Prague Czechia

**Keywords:** artificial intelligence, cervical cancer, chatgpt, deepseek, digital surgery, gemini, large language models

## Abstract

**Objective:**

Although large language models are increasingly used in clinical and research settings, the validity of the information they provide remains uncertain. This study aimed to evaluate the accuracy, consistency, and reliability of three large language models—ChatGPT 4.0, DeepSeek R1, and Gemini 2.0—in answering cervical cancer‐related questions based on the ESGO/ESTRO/ESP guidelines.

**Design:**

Prospective, comparative in silico benchmarking study.

**Setting:**

Fondazione Policlinico Universitario A. Gemelli, Rome, Italy. Population or Sample: Fifty questions derived from the ESGO/ESTRO/ESP (European Society of Gynaecologic Oncology/European Society for Radiotherapy and Oncology/European Society of Pathology) Guidelines for Cervical Cancer.

**Methods:**

Each question was submitted simultaneously to ChatGPT 4.0, DeepSeek R1, and Gemini 2.0, and re‐entered twice to assess response repeatability. Answers were evaluated for accuracy using a Global Quality Score (GQS) from 1 (poor) to 5 (completely accurate). Consistency (intra‐model response stability) and reliability (alignment with guidelines) were assessed using binary classification. Main Outcome Measures: Median GQS, percentage of GQS 5 responses, consistency between repeated answers, and reliability.

**Results:**

ChatGPT 4.0 achieved the highest performance, with 42% of responses rated GQS 5, followed by Gemini 2.0 (30%) and DeepSeek R1 (28%). DeepSeek R1 and Gemini 2.0 scored lower in median GQS (3.50) compared to ChatGPT 4.0 (4.00). Response consistency varied significantly, with ChatGPT 4.0 and DeepSeek R1 showing differences from Gemini 2.0 (*p* = 0.034 and *p* = 0.044, respectively). No significant difference was observed in reliability (*p* = 0.602).

**Conclusion:**

All models demonstrated suboptimal accuracy in aligning with clinical guidelines. ChatGPT 4.0 was the most accurate and consistent whereas DeepSeek R1 underperformed. Despite similar reliability across models, expert oversight remains essential to ensure safe clinical application and prevent misinformation.

## 1 Introduction

In the era of surgical innovations, the integration of new technologies in both healthcare and research represents a continuously evolving dynamic [1]. Until 2023, the potential of large language models remained largely unexplored. However, in the past two years, their use has grown exponentially [2]. These tools have become predominant not only as educational support but also as fundamental resources for information retrieval and assistance in clinical and scientific practices both as an aid in decision‐making and in information retrieval. However, their reliability remains unproven [3].

In the fields of education and medical research, large language models significantly contribute to improving access to knowledge by facilitating the extraction and interpretation of information [4]. However, their use must be approached critically. Although these models show great potential, they still exhibit limitations in reliability and contextual interpretation, making expert human supervision essential in their application.

Even though new large language models are emerging, there is still no clear consensus regarding their reliability or which one is currently the most accurate in generating responses [5]. In gynaecologic oncology, most of the available studies evaluate the capabilities of ChatGPT, the most commercially recognised large language model [6, 7]. Several studies have assessed its ability to pass medical exams [7, 8]. Additionally, ChatGPT has been evaluated for its role in assisting decision‐making in tumour boards [9, 10], or in answering questions about gynaecologic cancers in accordance with guidelines, with controversial results [3, 11].

Although the accuracy of large language models has not been validated yet, their use is widespread, even in scientific writing, raising ethical concerns and highlighting the need for updated control mechanisms in academic publishing as evidenced by retracted articles due to scientific misconduct [12]. To date, few studies have specifically evaluated the accuracy and completeness of large language models for treatment recommendations in cervical cancer [13], and all have focused exclusively on ChatGPT [3, 4].

Assuming that each large language model would achieve suboptimal accuracy compared to ESGO/ESTRO/ESP standards, the objective of this study was to assess the accuracy, consistency, and reliability of ChatGPT 4.0 and of new emerging large language models such as DeepSeek R1 and Gemini 2.0 in correctly answering questions related to the ESGO/ESTRO/ESP Guidelines for Cervical Cancer [14] without engineering prompts. Additionally, this study aims to compare the different large language models to determine which one provides the highest accuracy in responses concerning cervical cancer.

## 2 Methods

### 2.1 Large Language Models

ChatGPT 4.0, released by OpenAI (San Francisco, California, United States) in 2023, is based on a large language model architecture and operates through transformer deep learning, using fine‐tuning and reinforcement learning from human feedback techniques to enhance response quality and coherence. Its database has been updated until 2023, hence limiting direct knowledge of subsequent events. DeepSeek R1 (Hangzhou, People's Republic of China), launched in 2025, is designed to excel in natural language understanding and generation, with a particular focus on Chinese and English inputs. It leverages advanced retrieval augmented generation (RAG) methodologies to improve the accuracy of retrieved information. Its database has been updated until 2023. However, it can integrate real‐time data via the web. Gemini 2.0, developed by Google DeepMind (London, United Kingdom), officially released in 2024, can be distinguished by its multimodal capabilities, enabling the simultaneous processing of text, images, audio, and video. It uses a deep learning architecture to integrate various data types. The database of Gemini has been updated until 2023, although some versions can access more recent information via web‐based retrieval. Such models operate on cloud‐based platforms and are accessible through web or application programming interfaces, allowing users to engage with them for tasks ranging from automated writing to data analysis and complex problem‐solving.

### 2.2 ESGO/ESTRO/ESP Guidelines for Cervical Cancer

The ESGO/ESTRO/ESP (European Society of Gynaecologic Oncology/European Society for Radiotherapy and Oncology/European Society of Pathology) Guidelines for Cervical Cancer [14], published in 2023, provide evidence‐based recommendations for the diagnosis, management, and follow‐up of cervical cancer patients. These guidelines are an updated version of the 2018 ESGO/ESTRO/ESP Guidelines, incorporating the latest scientific evidence up to March 2022. In this study, these guidelines were used as a benchmark to assess the accuracy and clinical applicability of responses generated by the different large language models in the context of cervical cancer management.

### 2.3 Response Generation

A set of 50 questions was developed by two expert gynaecologic oncologists (NB, BC) based on the ESGO/ESTRO/ESP Pocket Guidelines for Cervical Cancer [14]. Throughout February 2025, these questions were simultaneously submitted to three large language models, namely ChatGPT 4.0, DeepSeek R1, and Gemini 2.0, with explicit instructions to generate responses in accordance with the latest ESGO guidelines, but without any prior training. All models were accessed via desktop browser interfaces using institutional accounts. Fifty clinically relevant questions were designed to test factual recall and application of ESGO guidelines content, avoiding hypothetical clinical decision‐making. All queries were performed using a zero‐shot prompting approach (i.e., without providing prior examples or contextual information). Each question was submitted twice to each large language model, using a different account for every session (six in total). To maintain an unbiased evaluation, a “single turn” approach was adopted by entering each query in a new independent workspace following a stateless interaction modality (each question was submitted in a new, independent chat session to prevent any memory or contextual carryover from previous interactions). The accuracy, consistency, and reliability of the responses were independently evaluated by two certified gynaecologic oncologists (NB and BC). In cases of discrepancies in judgement, a third gynaecologic oncologist reviewed the responses and resolved any disagreements (LL). Accuracy was assessed using a Global Quality Score (GQS) (Figure S1) ranging from 1 to 5, whereas consistency and reliability were evaluated through a binary classification system (Yes/No). Reliability was defined as the alignment of each model's response with the ESGO/ESTRO/ESP guidelines recommendations. Although no formal checklist was used, each answer was assessed against the relevant guidelines content by two independent gynaecologic oncology experts. Responses that contradicted or diverged from the guidelines were classified as “No”. Consistency (or repeatability) was evaluated by submitting each question twice in separate sessions for each model; if both responses fell into the same Global Quality Score category, the model was considered consistent for that question. If the two responses from the same large language models differed in accuracy, the final score was assigned based on the lower‐performing response. The consistency of large language model answers was evaluated by asking each enquiry twice in a new independent workspace. If the responses scored twice in the same category, the answer was considered positive in terms of reproducibility. If the two responses scored at different levels, it was accepted as negative repeatability (Table S1).

The final set of 50 questions was developed based on the ESGO/ESTRO/ESP Pocket Guidelines for Cervical Cancer, with the aim of covering all key recommendations. This number was selected to ensure comprehensive content coverage while maintaining feasibility for comparative evaluation across multiple models.

### 2.4 Statistical Analysis

Qualitative variables were summarised using absolute and percentage tables, whereas quantitative variables were reported as median and interquartile range (IQR). Comparisons of the Global Quality Score across LMM groups (ChatGPT 4.0, DeepSeek R1 and Gemini 2.0) were made using the Kruskal‐Wallis test, as appropriate for non‐normally distributed data. When the global test (Kruskal‐Wallis test) indicated a statistically significant difference among groups, pairwise post hoc comparisons were conducted using the Dunn test with Bonferroni correction for multiple testing.

Differences in consistency and reliability across LMM groups were assessed using the Chi‐squared test or Fisher's exact test, as appropriate. When a significant overall difference was found, pairwise post hoc Chi‐squared tests with Bonferroni correction were applied to identify which group comparisons accounted for the difference.

Inter‐rater reliability among evaluators before adjudication was assessed using Cohen's kappa (κ) for categorical variables and the Intraclass Correlation Coefficient (ICC, two‐way mixed model, absolute agreement) for quantitative variables.

A boxplot was drawn to show the difference in the Global Quality Score across LMM groups.

A *p* < 0.050 was considered statistically significant.

All statistical analyses were performed using the R statistical software (R, CRAN, 2024).

## 3 Results

### 3.1 Accuracy

**FIGURE 1:**
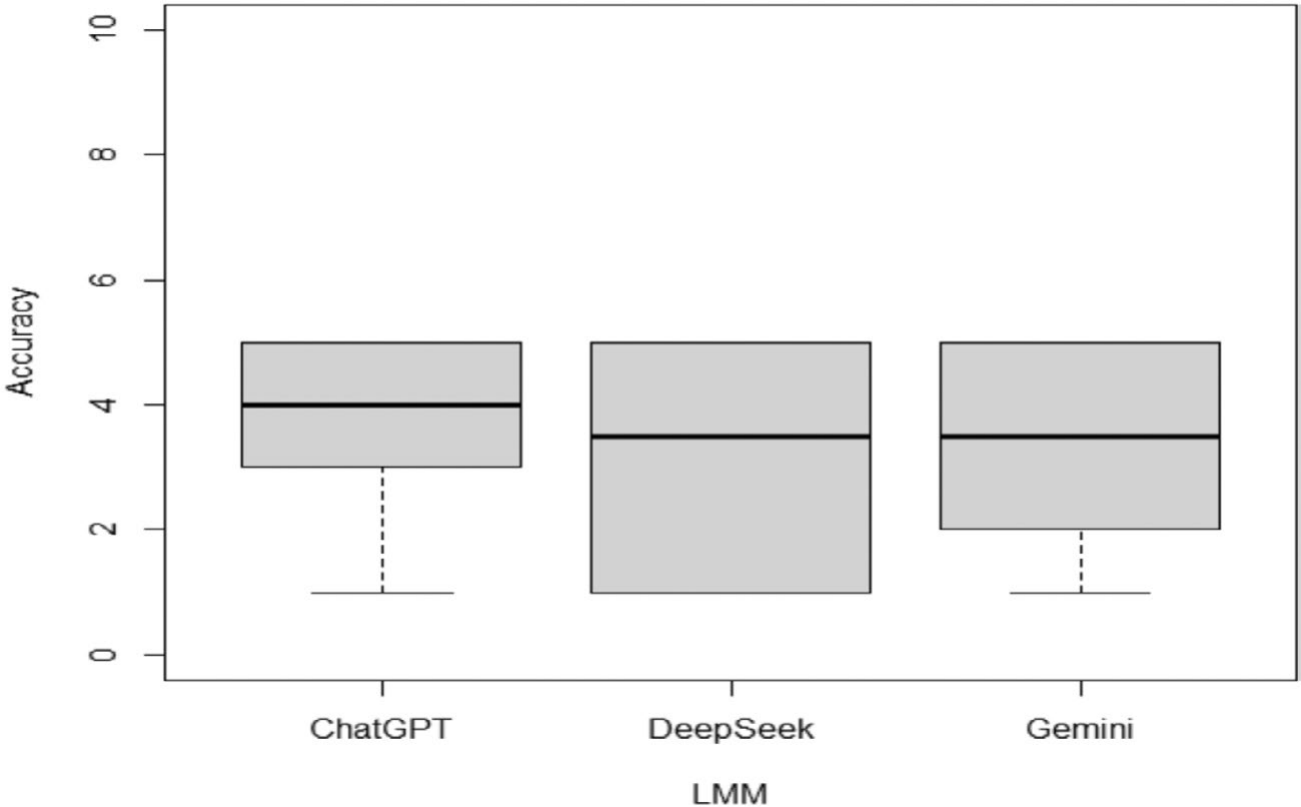
Boxplots for the accuracy of responses to questions related to ESGO/ESTRO/ESP Guidelines for Cervical Cancer using ChatGPT, DeepSeek, and Gemini. LLM: Large Language Models.

ChatGPT 4.0 provided 21 responses (42%) related to cervical cancer based on ESGO guidelines, which were completely accurate and satisfactory (GQS 5). Additionally, 12 responses (24%) were rated as GQS 4, 6 responses (12%) as GQS 3, 5 responses (10%) as GQS 2, and 6 responses (12%) as GQS 1. DeepSeek R1 achieved 14 responses (28%) rated as GQS 5 (completely accurate and satisfactory), whereas 16 responses (32%) were classified as poor quality (GQS 1). Gemini 2.0 produced 15 responses (30%) rated as GQS 5, whereas 10 responses (20%) were classified as good quality but scarce in content, and 11 responses (22%) were of moderate quality. The overall scoring of large language models' quality responses is summarised in Table 1 and Figure 1. On average, the accuracy of the responses, as measured by means of the GQS score, was of moderate quality. ChatGPT 4.0 demonstrated the highest accuracy. The differences in all scores were not statistically significant (*p* = 0.074), mainly due to the accuracy difference between ChatGPT 4.0 and DeepSeek R1 (*p* = 0.031) (Tables 1 and 2).

### 3.2 Consistency

**FIGURE 2:**
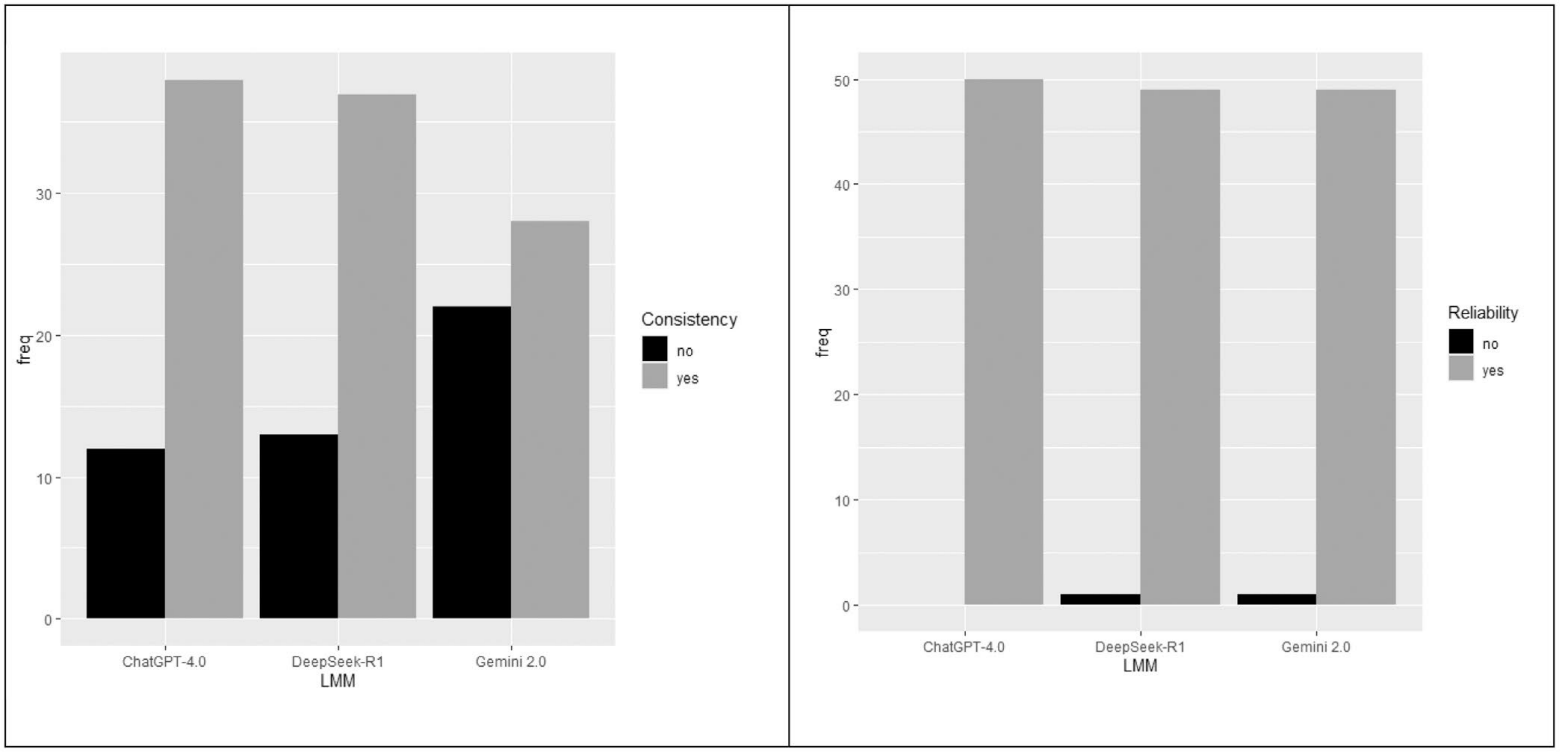
Barplots for consistency and reliability of responses to questions related to ESGO/ESTRO/ESP Guidelines for Cervical Cancer using ChatGPT, DeepSeek, and Gemini. LLM: Large Language Models.

The consistencies of ChatGPT 4.0 and DeepSeek R1 were similar, but both differed significantly from Gemini 2.0 (ChatGPT 4.0 vs. Gemini 2.0, *p* = 0.034; DeepSeek R1 vs. Gemini 2.0, *p* = 0.044) (Tables 1 and 2, and Figure 2).

### 3.3 Reliability

Regarding reliability, no statistically significant differences were observed between the large language models (*p* = 0.602), indicating that all models were equally reliable (Tables 1 and 2, and Figure 2).

### 3.4 Inter‐Rater Reliability Before Response Adjudication

Inter‐rater reliability among evaluators before adjudication was assessed using Cohen's κ for Consistency and Reliability, and the Intraclass Correlation Coefficient (ICC) for Accuracy (GQS).

Agreement between evaluators was almost perfect for Consistency (κ = 0.802 for ChatGPT 4.0; κ = 0.991 for DeepSeek R1; κ = 0.878 for Gemini 2.0) and for Reliability (κ = 0.921 for ChatGPT 4.0; κ = 0.892 for DeepSeek R1; κ = 0.889 for Gemini 2.0).

Inter‐rater reliability for Accuracy (GQS) before adjudication was assessed using the Intraclass Correlation Coefficient (ICC, two‐way mixed model, absolute agreement). The ICC for single measures was 0.942 for ChatGPT 4.0, 0.978 for DeepSeek R1, and 0.958 for Gemini 2.0, indicating excellent agreement between raters across all large language models.

Table 3 shows Intraclass Correlation Coefficient and Cohen's κ values.

## 4 Discussion

### 4.1 Main Findings

This study assessed the accuracy and quality of ChatGPT 4.0, DeepSeek R1, and Gemini 2.0 in generating responses based on *ESGO/ESTRO/ESP Guidelines* for Cervical Cancer [14]. ChatGPT 4.0 demonstrated the highest accuracy (42% GQS 5, median score 4), whereas DeepSeek R1 performed the weakest accuracy (28% GQS 5, median score 3.5) (*p = 0.031*). Gemini 2.0 showed moderate performance (Tables 1 and 3). A significant dichotomy in accuracy differences was shown, with ChatGPT 4.0 and Gemini 2.0 differing significantly from DeepSeek R1. The consistencies of ChatGPT 4.0 and DeepSeek R1 were similar. However, both differed significantly from Gemini 2.0. In spite of accuracy variations, all models presented similar relatively low reliability (Table 3).

**TABLE 1:** Results of Kruskal‐Wallis, Chi‐squared, Fisher test and post hoc test.

	ChatGPT 4.0	DeepSeek R1	Gemini 2.0	*p*(Overall)	Post hoc test (bonferroni‐adjusted)	Comparison	Adjusted *p*
Accuracy (GQS), Median (IQR)	4.00 (3.00–5.00)	3.50 (1.00–5.00)	3.50 (2.00–5.00)	*Kkruskal‐Wallis test* *p =* 0.074	*Dunn's test (Bonferroni)*	ChatGPT 4.0 vs. DeepSeek R1	***p* = 0.031**
						ChatGPT 4.0 vs. Gemini 2.0	*p* = 0.135
						DeepSeek vs. Gemini 2.0	*p* = 0.418
Consistency/Repeatability No, *n* (%) Yes, *n* (%)	12 (24%) 38 (76%)	13 (26%) 37 (74%)	22 (44%) 28 (56%)	*Chi‐ squared test* *p =* 0.059	*Chi‐squared test with Yates' continuity correction*	ChatGPT 4.0 vs. DeepSeek R1	*p* = 0.817
						ChatGPT 4.0 vs. Gemini 2.0	***p* = 0.034**
						DeepSeek vs. Gemini 2.0	***p* = 0.044**
Reliability No, *n* (%) Yes, *n* (%)	0 50 (100%)	1 (2%) 49 (98%)	1 (2%) 49 (98%)	*Fisher test* *p =* 0.602	—	—	—

**TABLE 2:** Inter‐rater reliability using Cohen's κ and ICC (Intraclass Correlation Coefficient*)* for Consistency/Repeatability, Reliability, and Accuracy (GQS); CI:(Confidence interval).

Variables	Cohen's κ /ICC	ChatGPT 4.0	DeepSeek R1	Gemini 2.0
Accuracy (GQS)	ICC (95% CI)	0.942 (0.87–0.98)	0.978 (0.95–0.99)	0.958 (0.91–0.98)
Consistency/Repeatability	Cohen's κ (95% CI)	0.802 (0.65–0.91)	0.991 (0.98–1.00)	0.878 (0.77–0.95)
Reliability	Cohen's κ (95% CI)	0.921 (0.83–0.97)	0.892 (0.79–0.95)	0.889 (0.78–0.95)

**TABLE 3:** Percentages for quality responses by ChatGPT 4.0, DeepSeek R1, and Gemini 2.0 to questions related to ESGO/ESTRO/ESP Guidelines for Cervical Cancer. GQS: Global Quality Score (scores ranging from 1 to 5, from poor [1] to excellent [5]).

GQS	Poor quality	Generally poor quality	Moderate quality	Good quality	Excellent quality
ChatGPT 4.0	6 (12%)	5 (10%)	6 (12%)	12 (24%)	21 (42%)
DeepSeek R1	16 (32%)	6 (12%)	3 (6%)	11 (22%)	14 (28%)
Gemini 2.0	10 (20%)	4 (8%)	11 (22%)	10 (20%)	15 (30%)

Importantly, DeepSeek recommended hyperthermic intraperitoneal chemotherapy (HIPEC) in a context where ESGO guidelines advise against it, posing a potential safety risk.

### 4.2 Comparison With the Published Literature

The evaluation of ChatGPT 4.0, DeepSeek R1, and Gemini 2.0 in providing responses to cervical cancer‐related enquiries, based on ESGO guidelines, highlights varying levels of accuracy and reliability among large language models.

These findings align with previous literature reports that assessed large language models in gynaecologic oncology [2]. Hermann et al. found that ChatGPT accurately provided responses on cervical cancer prevention and survivorship, but was less reliable for diagnosis and treatment [4]. Likewise, Yurtcu et al. noted that ChatGPT demonstrated a high reproducibility rate (93.2%) in answering general cervical cancer‐related FAQs, but exhibited a decline in accuracy when assessed against guidelines [3].

Additionally, a study by Finch et al. compared ChatGPT to NCCN guidelines in ovarian cancer management and found that ChatGPT 4 provided a greater proportion of complete responses when prompted as compared to non‐prompted answers [11]. Anastasio et al. demonstrated that although large language models such as ChatGPT (60%) and Bard (43%) provided useful information, physician responses remained superior in terms of quality and accuracy (86.7%) [15]. To date, this study was the only one, that investigated another large language model in gynaecologic oncology, showing that Bard, consistent with the findings of the present study, exhibited lower accuracy than ChatGPT, similar to Gemini 2.0 and DeepSeek‐R1 [15]. Several studies compared the performance of ChatGPT in passing official medical certification exams, reporting a mean accuracy of 61.1% (95% CI: 56.1%–66.0%) [7, 16, 17, 18]. A recent study specifically evaluated the ability of ChatGPT to correctly answer the Gynaecological Endoscopic Surgical Education and Assessment (GESEA) Level 1–2 knowledge tests. ChatGPT 3.5 achieved an overall accuracy of 59%, with 64% of responses providing comprehensive explanations. Notably, the model performed better on GESEA Level 1 questions (64% accuracy) compared to Level 2 (54% accuracy), suggesting a decline in performance as question difficulty increased [8]. The increasing reliance on large language models in academic writing raises concerns about their ability to generate human‐like content, which may go undetected by reviewers. A recent study in gynaecologic oncology found that reviewers could well distinguish between human‐ and ChatGPT‐generated abstracts only half of the time. Accuracy improved with greater academic experience and familiarity with artificial intelligence, highlighting the challenges of detecting generated scientific writing [6].

To date, no studies have assessed the accuracy of DeepSeek in clinical decision‐making or consultative support in health care. In contrast, existing evidence confirms that ChatGPT 4.0 outperforms Gemini 2.0. A recent study by Tarris et al. demonstrated that ChatGPT provided more accurate and consistent responses, particularly in multiple‐choice scenarios, whereas Gemini showed greater variability and slightly lower overall performance [19]. An additional study assessed GPT 4.0 and Gemini 2.0 in diagnosing acne and rosacea from clinical images. GPT 4.0 demonstrated high diagnostic accuracy, with 93% correct diagnoses, 93.0% sensitivity, and 97.7% specificity, whereas Gemini 2.0 correctly identified only 21% of cases [20].

### 4.3 Strengths and Limitations

This study is the first to analyse the accuracy of Gemini 2.0 and DeepSeek R1 in responding to questions related to gynaecologic oncology, and it has several strengths, which enhance the validity of its findings. First, the simultaneous administration of the same set of questions to all three AI models (ChatGPT 4.0, DeepSeek R1, and Gemini 2.0) ensured a controlled comparison, minimising the influence of external variables such as updates or algorithmic changes over time. Additionally, different accounts were used for each platform to prevent potential biases related to previous interactions or stored data, allowing for a more objective assessment of each model's response patterns.

The instruction to base responses on the most recent ESGO guidelines was intended to standardise the output. However, this approach may have inadvertently introduced bias in the reliability assessment, as it could have influenced how the models structured their answers rather than allowing for a completely independent evaluation. Notably, a pattern was observed in ChatGPT 4.0 where the second response tended to be shorter, yet more specific, raising questions about the possible session‐level variability.

Additionally, DeepSeek R1 presented significant limitations in terms of practical usability, since its servers were frequently unavailable for consecutive days, making it a less viable option for daily use. However, unlike the other models, DeepSeek R1 provided broader responses and uniquely cited multiple studies alongside ESGO guidelines, demonstrating a different referencing approach as compared to ChatGPT 4.0 and Gemini 2.0.

Nonetheless, this study has several limitations. One limitation is that the large language models evaluated had different knowledge cut‐off dates, which may have influenced their performance. The accuracy, consistency, and reliability of the responses were independently evaluated by two certified gynaecologic oncologists (NB and BC), which represents a major limitation due to the inherent subjectivity of expert judgement. The set of 50 questions was developed internally by two expert gynaecologic oncologists and was not externally validated, which may limit generalisability and reproducibility. Although we prioritised clinical relevance and adherence to ESGO/ESTRO/ESP guidelines, the absence of external validation introduces a potential source of bias. In addition, even though newer and more advanced AI models are publicly available, they were not evaluated in this study, and as a result, our conclusions are limited to the 2025 model versions tested. Additional limitations include possible question‐set bias, the use of ordinal‐scale statistics which may oversimplify complex outputs, and the omission of prompt‐engineering strategies, which could have potentially optimised model performance.

### 4.4 Implications

The findings of this study emphasise the need for further research to optimise the integration of large language models in gynaecologic oncology clinical practice while also addressing key ethical considerations. Although artificial intelligence‐powered large language models such as ChatGPT 4.0, DeepSeek R1, and Gemini 2.0 demonstrate potential in providing evidence‐based responses, their inconsistencies highlight the necessity for specialised models for oncologic applications. Future efforts should focus on developing domain‐specific large language models fine‐tuned on gynaecologic oncology datasets, using structured prompting strategies and validated against guideline‐based reference standards to ensure accuracy and clinical applicability. To ensure real‐world utility, these models should be capable of addressing clinical queries by not only adhering to international guidelines but also incorporating patient‐specific clinical variables for more tailored and context‐aware recommendations. Additionally, research should focus on validating the reliability of large language model‐generated references. Rigorous evaluation of such references is necessary to ensure that artificial intelligence‐assisted information retrieval aligns with high scientific and ethical standards.

From an ethical standpoint, the increasing reliance on artificial intelligence in clinical decision‐making and academic writing raises concerns about accountability, transparency, and potential biases embedded in such models. Physicians and researchers must critically assess artificial intelligence‐generated content to prevent the dissemination of misinformation and avoid over‐reliance on tools that may lack contextual awareness. Ethical guidelines should be developed to regulate the responsible use of large language models in medical research, ensuring that their implementation supports, rather than replaces, expert clinical judgement. Additionally, longitudinal studies assessing the impact of large language models in real clinical decision‐making scenarios, including tumour board discussions and patient counselling, could well provide deeper insights into their practical utility and limitations.

## 5 Conclusion

This study is the first to compare ChatGPT 4.0, DeepSeek R1, and Gemini 2.0 in answering cervical cancer–related questions based on the ESGO/ESTRO/ESP guidelines. In line with our initial hypothesis, all three large language models demonstrated relatively low accuracy in responding to guideline‐based questions concerning cervical cancer. ChatGPT 4.0 demonstrated the highest accuracy whereas DeepSeek R1 performed the weakest accuracy, with Gemini 2.0 in between. Although accuracy and consistency varied, all models showed similar relatively low reliability, emphasising that they should only be used as supplementary tools rather than primary decision‐making aids to date.

Future research should focus on developing artificial intelligence models specifically trained for gynaecologic oncology, improving structured prompting, and validating large language model‐generated references to prevent misinformation. Even though large language models hold great potential, expert supervision remains essential.

## Author Contributions

Study design: M.P., D.Q., N.B.; Manuscript drafting: M.P., C.I., N.M.; Data collection: N.M., C.I., C.C., M.C., A.R., B.C., N.B.; Statistical analysis: A.C.; Senior supervisors: L.L., B.C., J.M., A.F., F.F., D.C., D.Q.; Critical revision of the manuscript: J.M., L.L., B.C., A.F., F.F., D.C., D.Q., N.B., N.B. and D.Q. were guarantors for the publication. All authors have read and commented on the previous version of the article. All authors approved the final version of the article prior to submission.

## Funding

The authors have nothing to report.

## Ethics Statement

Since no human participants or animal subjects were involved in this study, approval from an institutional review board or local ethics committee was not required.

## Conflicts of Interest

The authors declare no conflicts of interest.

## Supporting information


**Figure S1:** Global Quality Score.

**Table S1:** Lists of questions and scores.

## Data Availability

The data that support the findings of this study are available from the corresponding author upon reasonable request.
