# Supplementary material for: Assessing the Accuracy of Large Language Models on European Guidelines for Cervical Cancer: An In Silico Benchmarking Study
Source: BJOG. 2025 Nov 24;133(4):771–8. doi: 10.1111/1471-0528.70095 (PMC12884235; doi:10.1111/1471-0528.70095)
Supplement: Supplementary file 1 — Figure S1: Global Quality Score. [file BJO-133-771-s002.docx]

Supplement Online A

| **Category assested** | **Tool** | **Score range** | **Interpretation** |
| --- | --- | --- | --- |
| Accuracy | Global Quality Score (modified)   1. Poor quality of the answer: most information missing 2. Generally poor quality of the answer: some information listed but many important topics missing 3. Moderate quality of the answer: suboptimal, some important information is adequately discussed but others poorly discussed, 4. Good quality of the answer: most of the relevant information is listed, but some topics not covered 5. Excellent quality of the answer | 1-5 | Higher values indicate higher quality |
